# Curcumin Inhibits Transforming Growth Factor-β1-Induced EMT via PPARγ Pathway, Not Smad Pathway in Renal Tubular Epithelial Cells

**DOI:** 10.1371/journal.pone.0058848

**Published:** 2013-03-27

**Authors:** Rui Li, Yunman Wang, Yujun Liu, Qijing Chen, Wencheng Fu, Hao Wang, Hui Cai, Wen Peng, Xuemei Zhang

**Affiliations:** 1 Department of Pharmacology, School of Pharmacy, Fudan University, Shanghai, China; 2 Department of Nephrology, Putuo Hospital, Shanghai University of Traditional Chinese Medicine, Shanghai, China; 3 Renal Division, Department of Medicine, Emory University School of Medicine, Atlanta, Georgia, United States of America; 4 Renal Section, Atlanta Veteran Administration Medical Center, Decatur, Georgia, United States of America; UAE University, Faculty of Medicine & Health Sciences, United Arab Emirates

## Abstract

Tubulointerstitial fibrosis (TIF) is the final common pathway in the end-stage renal disease. Epithelial-to-mesenchymal transition (EMT) is considered a major contributor to the TIF by increasing the number of myofibroblasts. Curcumin, a polyphenolic compound derived from rhizomes of Curcuma, has been shown to possess potent anti-fibrotic properties but the mechanism remains elusive. We found that curcumin inhibited the EMT as assessed by reduced expression of α-SMA and PAI-1, and increased E-cadherin in TGF-β1 treated proximal tubular epithelial cell HK-2 cells. Both of the conventional TGF-β1/Smad pathway and non-Smad pathway were investigated. Curcumin reduced TGF-β receptor type I (TβR-I) and TGF-β receptor type II (TβR II), but had no effect on phosphorylation of Smad2 and Smad3. On the other hand, in non-Smad pathway curcumin reduced TGF-β1-induced ERK phosphorylation and PPARγ phosphorylation, and promoted nuclear translocation of PPARγ. Further, the effect of curcumin on α-SMA, PAI-1, E-cadherin, TβR I and TβR II were reversed by ERK inhibitor U0126 or PPARγ inhibitor BADGE, or PPARγ shRNA. Blocking PPARγ signaling pathway by inhibitor BADGE or shRNA had no effect on the phosphorylation of ERK whereas the suppression of ERK signaling pathway inhibited the phosphorylation of PPARγ. We conclude that curcumin counteracted TGF-β1-induced EMT in renal tubular epithelial cells via ERK-dependent and then PPARγ-dependent pathway.

## Introduction

Renal fibrosis, characterized by accumulation of fibroblasts and excessive matrix proteins along with loss of functioning nephrons, is a major pathological feature of progressive kidney disease. Tubulointerstitial fibrosis is considered the final common pathway of renal fibrosis. Recent studies have demonstrated that a critical step in the pathogenesis of tubulointerstitial fibrosis is Epithelial-mesenchymal transition (EMT) [1], a process whereby fully differentiated epithelial cells undergo transition to a mesenchymal phenotype. EMT causes a substantial increase in the number of myofibroblasts, one of main effector cells that contributes to the development of progressive renal fibrosis [2].

TGF-β is known as a major inducer of EMT. TGF-β1 induced EMT via Smad-dependent and Smad-independent pathways [3]. Through the Smad mediated pathway, TGF-β signals are transduced by transmembrane serine/threonine kinase type II and type I receptors (TβR II and TβR I) and intracellular mediators Smads [4]. In the non-Smad signaling pathway, TGF-β receptors interact with the MAPK pathway [5]. There are also reports that Peroxisome proliferator-activated receptor-γ (PPAR-γ) activation exerts antiproliferative and antifibrotic effects via the modulation of TGF-β1-mediated pathways [6].

PPAR-γ is a member of the nuclear receptor family of transcription factors. Ligands for PPAR-γ include a variety of natural and synthetic compounds. Synthetic ligands are often used as insulin sensitizing agents for treatment of type 2 diabetes [7]. Studies have demonstrated that PPARγ agonists exert protective effects in the models of renal diseases [8], [9]. PPARγ agonists rosiglitazone significantly attenuated glomerulosclerosis, tubulointerstitial expansion and collagen IV deposition in the apolipoprotein E knockout mouse [10]. Troglitazone, another PPARγ agonists, also attenuated renal interstitial fibrosis and inflammation in the unilateral ureteral obstruction's animal (UUO) [11], a classic renal fibrosis model.

Curcumin [1,7-bis(4-hydroxy-3-methoxyphenyl)-1,6-heptadience-3,5-dione] is a natural polyphenolic compound derived from the root of curcuma longa that has been widely used in India for medical, culinary and other purposes. A large body of evidence from *in vitro* and *in vivo* studies of both animals and human have indicated that curcumin exhibits a variety of biological effects such as anti-oxidant, anti-inflammatory, anti-tumor and wound healing properties [12]. In particular, some recent studies have shown that curcumin has anti-fibrotic effect in liver, lung and cystic fibrosis [13], [14], [15]. In UUO rat kidney fibrosis model curcumin has been reported to inhibit the renal interstitial inflammation and fibrosis by inhibiting of the NF-κB-dependent pathway [16]. In immortalized rat kidney interstitial fibroblasts (NRK/49F), curcumin attenuated TGF-β-induced fibrosis through down-regulation of TβR II [17].

Curcumin has been reported to activate PPAR-γ as well [18], but it is unclear the effect is depend on binding to the receptor of PPAR-γ [18] or is the result of indirect effects [19]. Thus, we hypothesized that curcumin may inhibit renal fibrosis as assessed by EMT through PPARγ pathways in the TGF-β signaling. On the other hand, researches on the anti-renal fibrosis effect of curcumin have been concentrated on the mediating role of Smad pathway and little is known about curcumin's effects through the non-Smad pathway such as MAPK, and whether there is any cross talk between MAPK and PPARγ is still elusive. Here, we reported the effect of curcumin on TGF-β1-induced EMT in renal tubular epithelial cells, and its underlying mechanisms related to non-Smad ERK1/2 and PPARγ pathways.

## Materials and Methods

### Cell culture and treatment

Human proximal tubular epithelial cells (HK-2 cells) were cultured in RPMI 1640 containing 2000 mg/L NaHCO3, supplemented with 10% FBS, 100 units/ml penicillin and 100 ug/ml streptomycin in an atmosphere of 5% carbon dioxide and 95% air at 37°C. Normal Rat Kidney(NRK-52E) proximal cells were cultured in DMEM containing 2000 mg/L NaHCO3, supplemented with 10% FBS, 100 units/ml penicillin and 100 ug/ml streptomycin in an atmosphere of 5% carbon dioxide and 95% air at 37°C.

At about 90% confluent, the cells were trypsinized by treatment with trypsin-EDTA and seeded in 6-well plates, grown to confluence, and rested in medium without FBS overnight.

### Reagents

Curcumin [1,7-bis(4-hydroxy-3methoxyphenyl)-1,6-heptadiene-3,5-dione] was purchased from Sigma (St. Louis, MO, USA). Stock solutions of curcumin were prepared in dimethyl sulfoxide and used at a final concentration ≤0.1% (v/v). Recombinant Human transforming growth factor beta 1 (TGF-β1) were purchased from R&D Systems (USA). Antibodies to phospho-smad2, smad2, phospho-smad3, smad3, phospho-ERK1/2, ERK 1/2, PPARγ and TGF-β receptor II were purchased from Cell Signaling Technology (USA). Antibodies to p-PPARγ were purchased from Bioworld (USA). TGF-β receptor I was purchased from Santa Cruz Biotechnology (CA, USA). Antibodies to PAI-1, E-cadherin and α-SMA were purchased from Epitomics (USA). BCA Protein Assay Kit was purchased from Shanghai Biocolor BioScience & Technology Company (China).

### Western blot analysis

At the time of harvest, cells were washed twice with PBS (135 mM, 2.7 mM KCL, 1.5 mM KH_2_PO_4_, 8 mM K_2_HPO_4_), homogenized in RIPA lysis buffer (150 mM NaCl, 1% Triton X-100, 0.1% SDS, 50 mM Tris-HCl pH 7.4, 1 mM EDTA, 1 mM PSFM). The supernatants were collected after centrifugation at 10,000×g at 4°C for 15 min. Protein concentration was determined using a BCA Protein Assay Kit. Equal amounts of protein were boiled at 95°C for 5 min after the addition of 5× Laemmli buffer. Protein samples were separated by 10% SDS gel electrophoresis (SDS-PAGE) and transferred to PVDF membranes. After blocking with 5% dry milk in Tris-buffered saline with 0.1% Tween (TBST) at room temperature for 2 h, the membranes were incubated with primary antibodies at 4°C overnight. After being washed in TBST three times, the membranes were incubated with horseradish peroxidase (HRP)-conjugated secondary antibody at room temperature for 1 h. The signals were visualized using the enhanced chemiluminescent (ECL) substrate.

### Stable shRNA mediated repression of PPARγ in HK-2 cells

Human PPARγ expression in HK-2 cells was silenced by shRNA interference. The lentiviral mediated shRNA against PPARγ (NM_138712.3) system was purchased from Shanghai Sunbio Medical Biotechnology. An adopted non-silencing control shRNA sequence (TTCTCCGAACGTGTCACGT) that was not complementary to any human gene was used as a control shRNA. Lentiviruses were prepared in HEK293T cells followed by infecting HK-2 cells. Cells were selected using 0.5 μg/ml puromycin and subjected to western blot to test the expression level of PPARγ in the infected HK-2 cells.

### Immunofluorescent staining and ﬂuorescent microscopy

HK-2 cells grown on cover slips were washed with cold PBS three times and fixed in 5% paraformaldehyde for 25 minutes. The cells were then extensively washed three times each for 10 minutes with PBS and permeabilized with 0.1% TritonX-100 for 5 minutes. After blocking in 5% bovine serum albumin (BSA) in PBS buffer for 1 hour at room temperature, cells were incubated with the anti-PPARγ antibody at 4°C overnight with gentle rocking. Cells were next washed three times in PBS and stained with Cy-2-conjugated secondary antibody at room temperature for 2 hours. Stained cells were mounted with antifade mounting medium on slides and viewed with a ﬂuorescence microscope.

### Statistical Analysis

The data are shown as mean±SD. Statistical significance was assessed using a Student,s paired t test when there were only two groups involved. P<0.05 was considered statistically significant. All data analyses were performed using the SPSS software.

## Results

### 1. Curcumin inhibited the expression of α-SMA and PAI-1 and increased E-cadherin in TGF-β1 treated HK-2 cells

**Figure 1:**
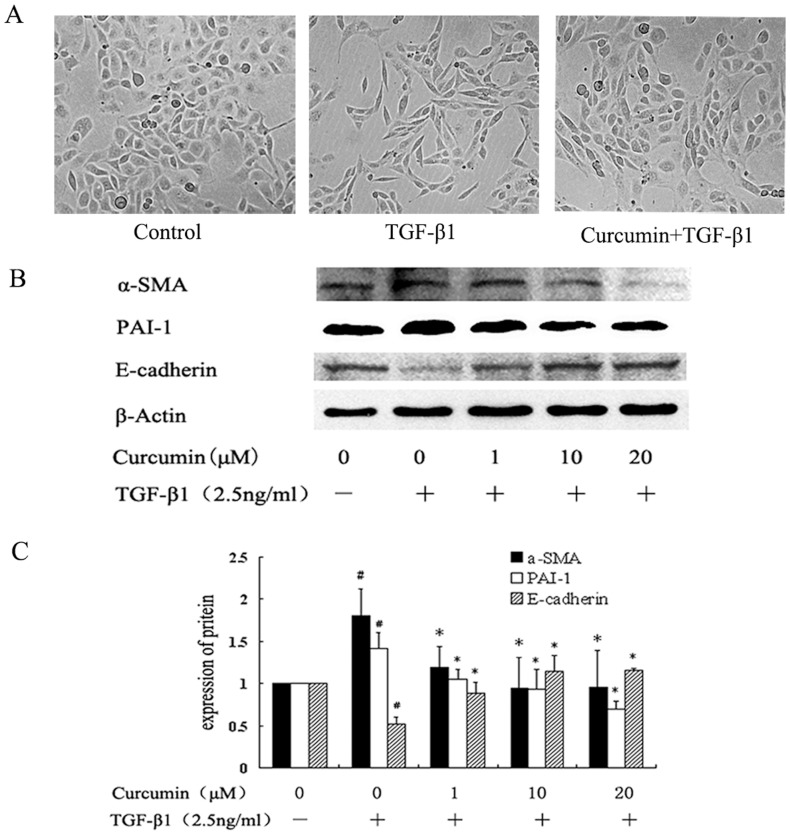
Effects of curcumin on morphology and the expression of α-SMA and PAI-1 and E-caherin in TGF-β1-induced HK-2 cells. HK-2 cells were incubated with curcumin at the indicated dose for 12 hours followed by treatment with TGF-β1 for 36 hours. (A) Effects of 10 μM of curcumin on morphology of HK-2 cells (B) Western blotting analyses for α-SMA, E-caherin and PAI-1 expression in HK-2 cells. (C) Graphical presentation of the relative expression of α-SMA, E-caherin and PAI-1. The values were represented as the density of α-SMA, E-caherin or PAI-1 vs β**-**actin (%). The mean ± SD (n = 3) of values were obtained from densitometric analysis of all individual experiments. *p<0.05 vs TGF-β1 alone.

As shown in **Fig.** **1**, the amount of α-SMA and PAI-1 proteins were very low while the epithelial cell marker, E-cadherin was prominent in normal HK-2 cells (**Fig.** **1** **b**). TGF-β1 induced a significant increase in α-SMA and PAI-1 and decrease in E-cadherin (**Fig.** **1** **b & c**). Curcumin down-regulated α-SMA and PAI-1 and up-regulated E-cadherin (**Fig.** **1** **b & c**).

### 2. Curcumin attenuated the TGF-β1-induced expression of TGF-β receptor type I (TβR I) and TGF-β receptor type II (TβR II) in HK-2 cells

**Figure 2:**
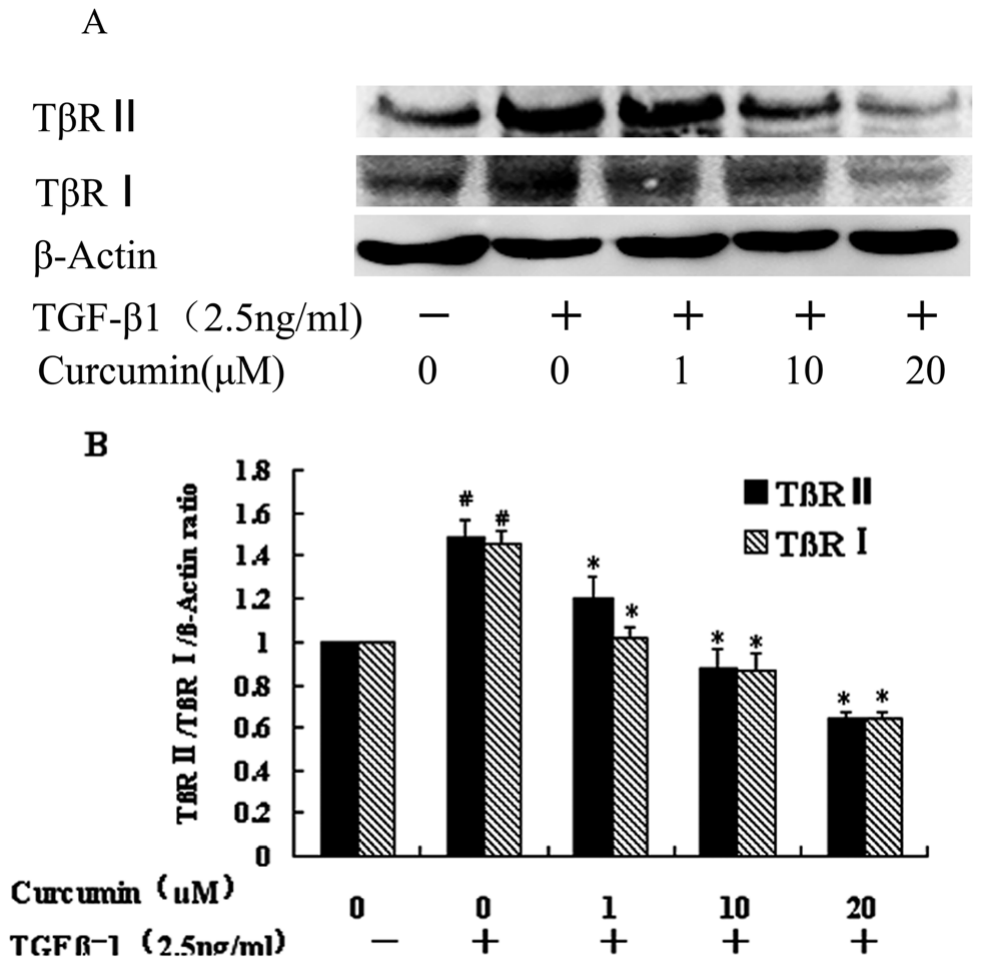
Effects of curicumin on TGF-β1-induced TβR I and TβR II expression. HK-2 cells were incubated with curcumin at the indicated dose for 12 hours followed by treatment with TGF-β1 for 36 hours. (A) Western blotting analyses for TβR I and TβR II expression in HK-2 cells. (B) Graphical presentation of the relative expression of TβR I and TβR II. The values were represented as the density of TβR I or TβR II vs β-actin (%). The mean ± SD (n = 3) of values were obtained from densitometric analysis of all individual experiments. ^#^P<0.05 vs. control alone, *P<0.05 vs TGF-β1 alone.

Binding to the TGF-β receptor type II (TβR II) is the first step of TGF-β1 signaling, and the TβR II in turn phosphorylates TGF-β receptor type I (TβR I) [20]. To determine whether the anti EMT effect of curcumin is targeting TGF-β1-related signaling, we studied the effects of curcumim on TβR II and TβR I. As shown in **Fig.** **2**, curcumin decreased both TβRI and TβR II expression in HK-2 cells.

### 3. Effects of curcumin on TGF-β1-induced Smad2 and Smad3 phosphorylation in HK-2 cells

We then studied the effects of curcumin on Smad2 and Smad3, two important mediators of the TGF-β/Smads signaling, which has been known to play a major role in TGF-β1-induced EMT [21].

**Figure 3:**
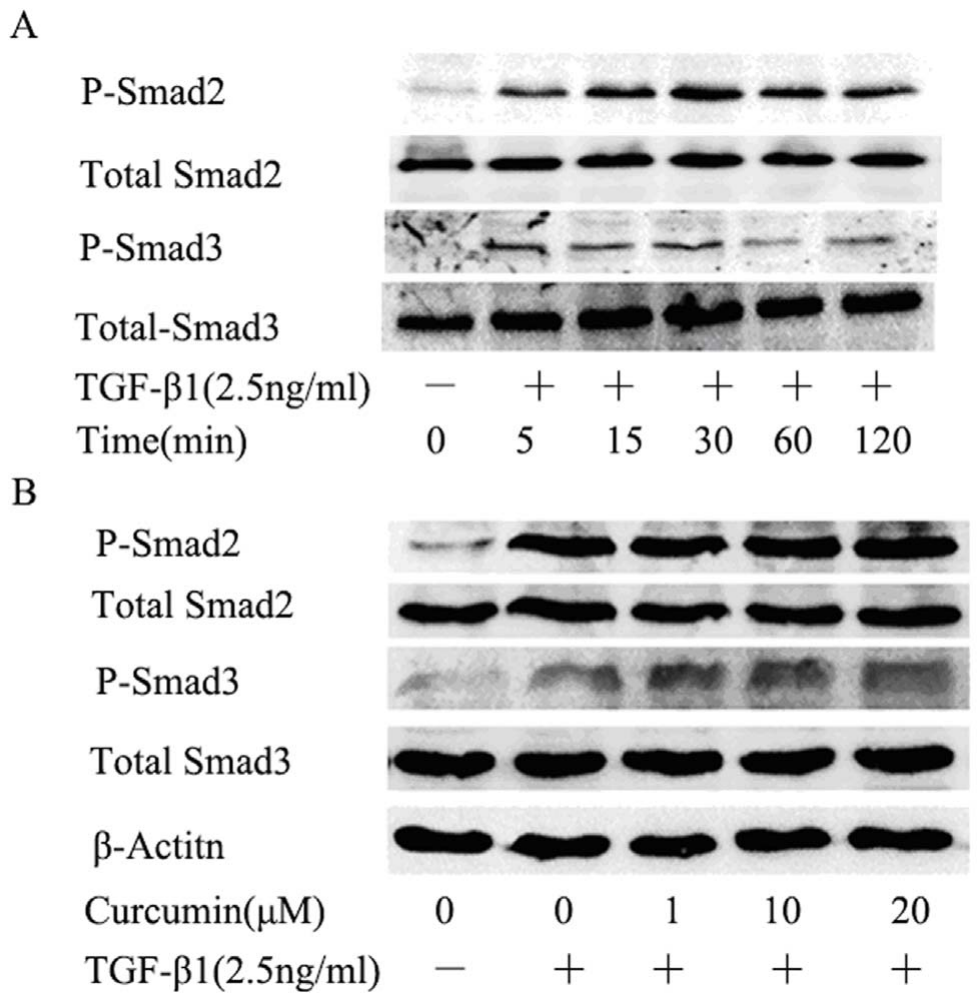
Effects of curcumin on TGF-β1-induced Smad2 and Smad3 phosphorylation in HK-2 cells. (A) HK-2 cells were stimulates with TGF-β1 (2.5 ng/ml ) for the indicated times, and the phosphorylation of Smad2 and Smad3 were analyzed by western blotting. (B) Western blotting analyses for p-Smad2 and p-Smad3 expression in HK-2 cells. HK-2 cells were incubated with curcumin at the indicated doses for 12 hours followed by treatment with TGF-β1 for 30 minutes.

Smad2 and Smad3 were phoshorylated at 5 min and the phosphorylations reached the peak levels at 30 min (**Fig.** **3a**). Pretreatment with curcumin for 12 hours and following by treatment with TGF-β1 for 30 minutes has no effect on phosphorylation of Smad2 and Smad3 (**Fig.** **3b**). These data indicated that the inhibition of TGF-β1-induced EMT by curcumin is not through TGF-β/Smads signaling pathway.

### 4. Curcumin reduced TGF-β1-induced ERK phosphorylation in HK-2 cells

**Figure 4:**
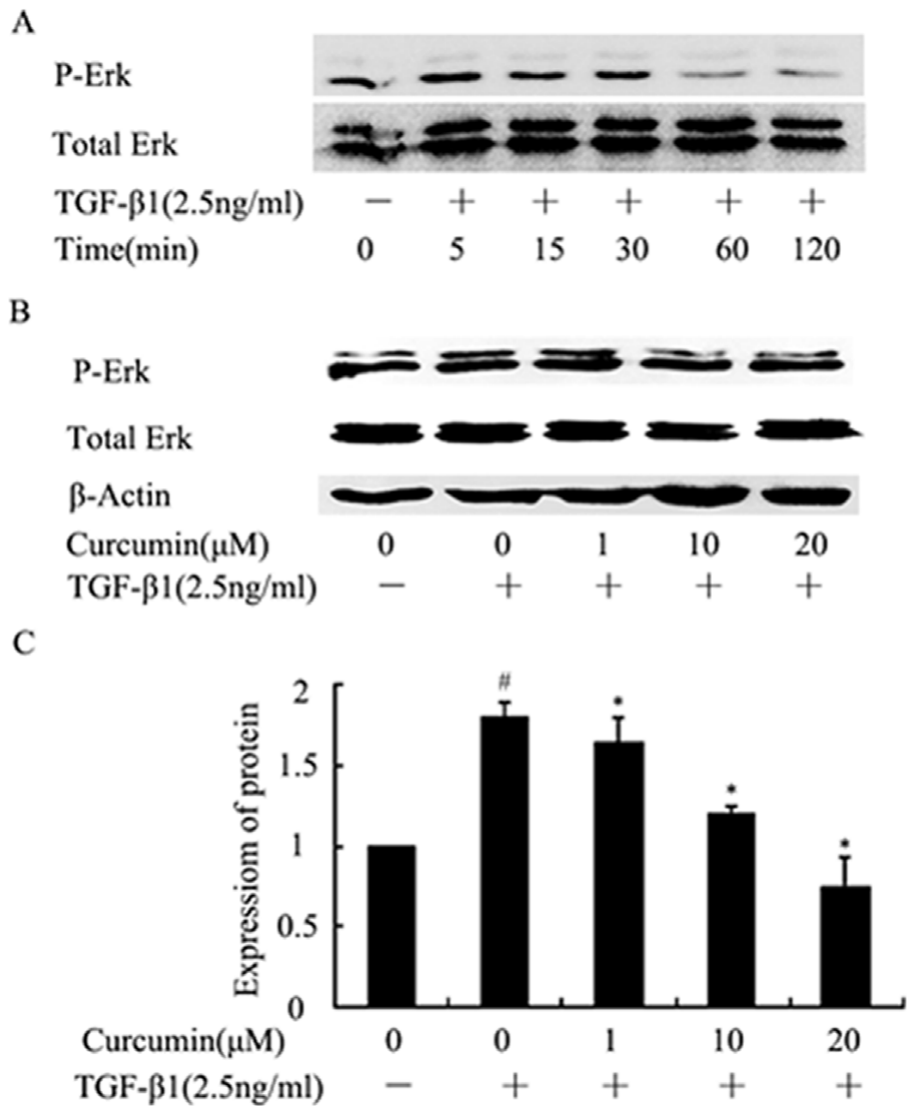
Effects of curcumin on TGF-β1-induced ERK phosphorylation in HK-2 cells. (A) HK-2 cells were stimulates with TGF-β1 (2.5 ng/ml ) for the indicated times, and the phosphorylation of ERK was analyzed by western blotting. (B) Western blotting analyses for p-ERK expression in HK-2 cells. HK-2 cells were incubated with curcumin at the indicated doses for 12 hours followed by treatment with TGF-β1 for 30 minutes. (C) Graphical presentation of the relative expression of pERK. The values were represented as the density of p-ERK vs total ERK (%). The mean ± SD (n = 3) of values were obtained from densitometric analysis of all individual experiments. ^#^P<0.05 vs. control alone, *P<0.05 vs TGF-β1 alone.

Our studies showed that curcumin had little effect on TGF-β/Smads signaling. Since the activation of TβRI and TβR II can lead to the activation of other EMT associated signaling pathways, such as ERK [22], we examined the influence of curcumin on ERK signaling. ERK was phoshorylated at 5 min and reached the peak levels at 30 min post TGF-β1 treatment (**Fig.** **4a**). Pretreatment with curcumin for 12 hours and following by treatment with TGF-β1 for 30 minutes decreased phosphorylation of ERK (**Fig.** **4b**). These data indicated that curcumin may inhibit TGF-β1-induced EMT through ERK-related signaling pathway.

### 5. Curcumin reduced TGF-β1-induced PPARγ phosphorylation and promoted nuclear translocation of PPARγ

**Figure 5:**
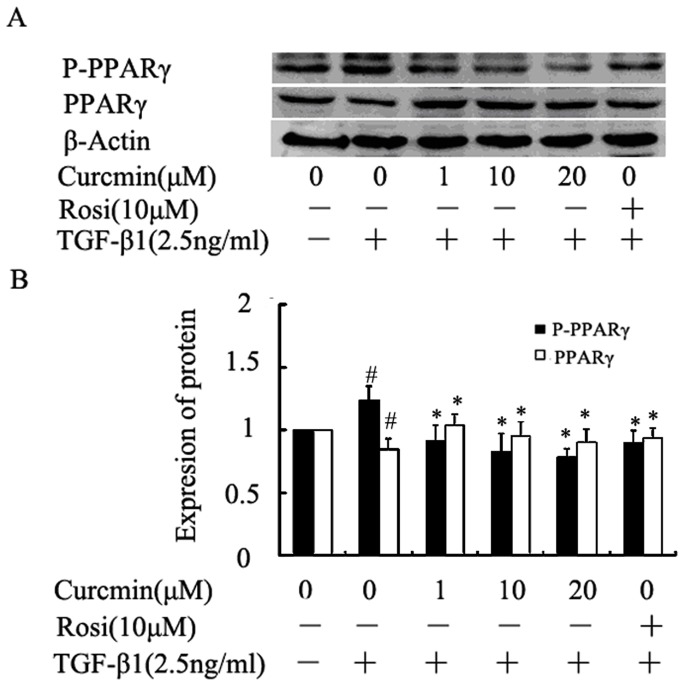
Curcumin reduced TGF-β1-induced PPARγ phosphorylation and reversed the reduction of PPARγ. HK-2 cells were incubated with curcumin at the indicated dose for 12 hours or rosiglitazone for 1 hour followed by treatment with TGF-β1 for 36 hours. (A) Western blotting analyses for PPARγ and p-PPARγ expression in HK-2 cells. (B) Graphical presentation of the relative expression of PPARγ and p-PPARγ. The values were represented as the density of PPARγ and p-PPARγ vs β-actin (%). The mean ± SD (n = 3) of values were obtained from densitometric analysis of all individual experiments. ^#^P<0.05 vs control alone, *P<0.05 vs TGF-β1 alone.

Total PPARγ expression was reduced while its phosphorylation was increased after being treated with TGF-β1(**Fig.** **5**)**.** Pretreatment with curcumin for 12 hours increased PPARγ expression level and inhibited the phosphorylation of PPARγ. As expected, PPARγ agonist rosiglitazone increased PPARγ expression. We also found that rosiglitazone inhibited the phosphorylation of PPARγ. These results indicated that curcumin is a PPARγ agonist.

**Figure 6:**
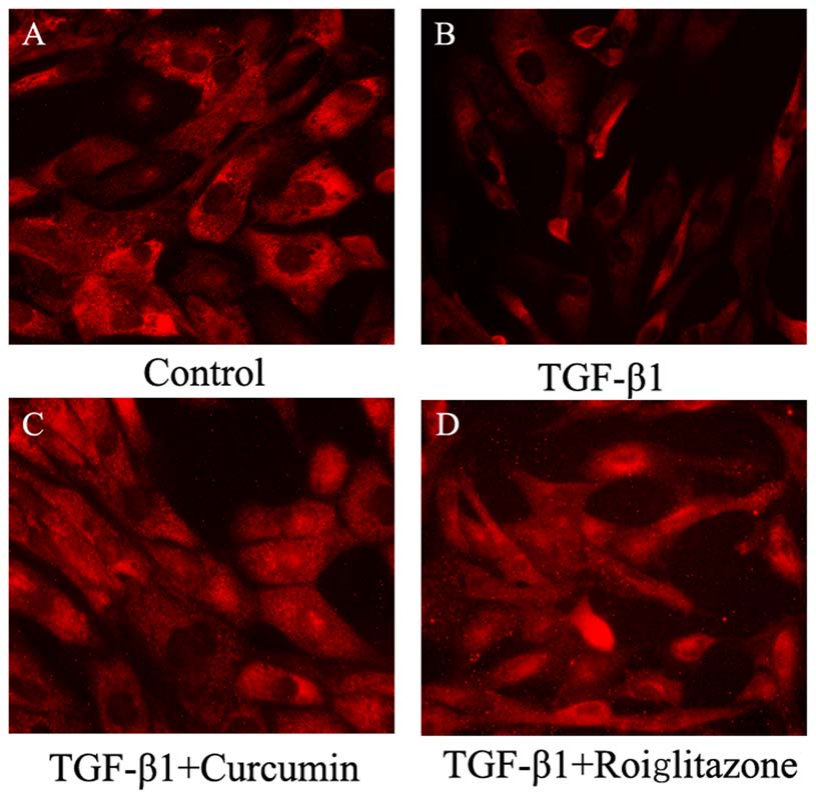
Effects of curcumin on nuclear translocation of PPARγ. HK-2 cells were pretreated with curcumin (10 μM) for 12 hours (C) or rosiglitazone (10 μM) for 1 hour (D) following by incubated with TGF-β1 for 36 hours. *Red for PPARγ.*

PPARγ is a nuclear transcription factor, which requires nuclear translocation. As shown in **Fig.** **6**, TGF-β1 decreased the expression of PPARγ, and curcumin increased the expression of PPARγ and promoted nuclear translocation of PPARγ in HK-2 cells. Rosiglitazone also promoted nuclear translocation of PPARγ.

### 6. ERK pharmacological inhibitor and PPARγ pharmacological inhibitor blocked the inhibitory effect of curcumin on TGF-β1-induced EMT of HK-2 cells

**Figure 7:**
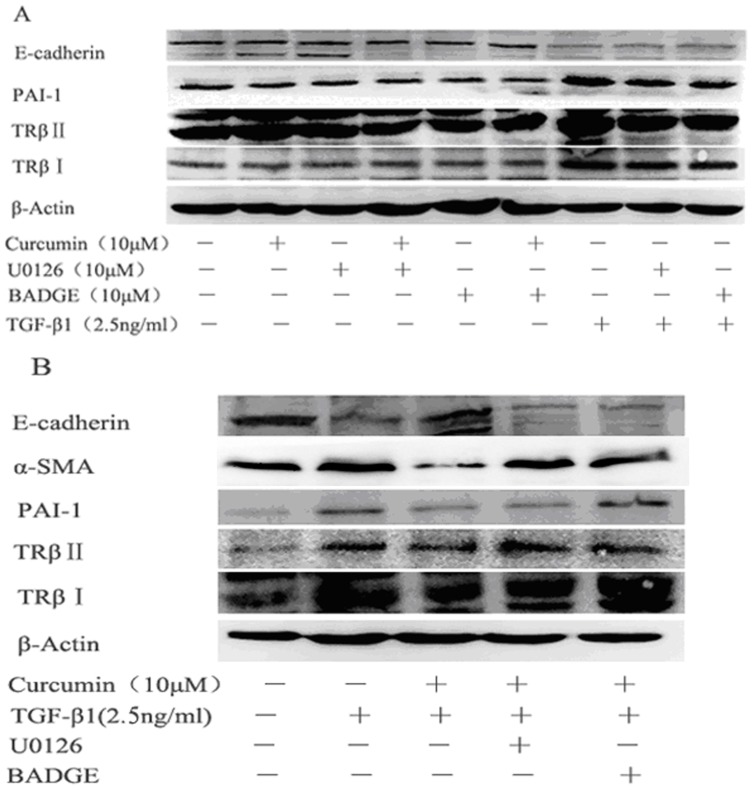
ERK1/2 inhibitor U0126 and PPARγ inhibitor BADGE blocked the effects of curcumin on TGF-β1-induced EMT in HK-2 cells. (A) Effects of U0126, BADGE on the expression of α-SMA, PAI-1, E-cadherin, TβR I and TβR II with or without TGF-β1. HK-2 cells were pretreated with curcumin (10 μM) for 12 hours followed by treatment with U0126 (10 μM) or BADGE (10 μM) for 36 hours or HK-2 cells were pretreated with U0126 (10 μM) or BADGE (10 μM) for 1 hour before addition of TGF-β1 (2.5 ng/ml) for 36 hours. (B) Effects of curcmin on the expression of α-SMA, PAI-1, E-cadherin, TβR I and TβR II with TGF-β1 and U0126 or BADGE. HK-2 cells were pretreated with curcumin (10 μM) for 12 hours at indicated dose followed by treatment with U0126 (10 μM) or BADGE (10 μM) for 1 hour before addition of TGF-β1 (2.5 ng/ml) for 36 hours.

To elucidate whether the inhibition of ERK and PPARγ signaling are necessary for the effect of curcumin on TGF-β1-induced EMT of HK-2 cells, we pretreated HK-2 cells with U0126, a pharmacologcal inhibitor of ERK, or BADGE, a pharmacologcal inhibitor of PPARγ. In order to exclude the influence of curcumin or inhibitors on the protein expression, we have designed a control experiment with or without TGF-β1. As shown in **Fig.** **7a**, in the absence of TGF-β1, curcumin, U0126, BADGE and curcumin combined with U0126, or BADGE did not have any effect on the protein levels of α-SMA, E-cadherin, PAI-1, TβRI and TβRII, and there was no significant difference compared with the control group. Similarly, in the presence of TGF-β1, U0126 or BADGE did not have any effect on establishment of model, and there was no significant difference compared with TGF-β1 treated group. Based on the above results, we studied the effects of curcumin with U0126 or BADGE on α-SMA, E-cadherin, PAI-1, TβR I and TβR II. The U0126 or BADGE treatment was shown to reverse the increases in α-SMA, PAI-1, TβR I and TβR II and the decreases in E-cadherin caused by curcumin (**Fig.** **7b**).

### 7. Effect of curcumin on TGF-β1-induced EMT in HK-2 cells is dependent on PPARγ

**Figure 8:**
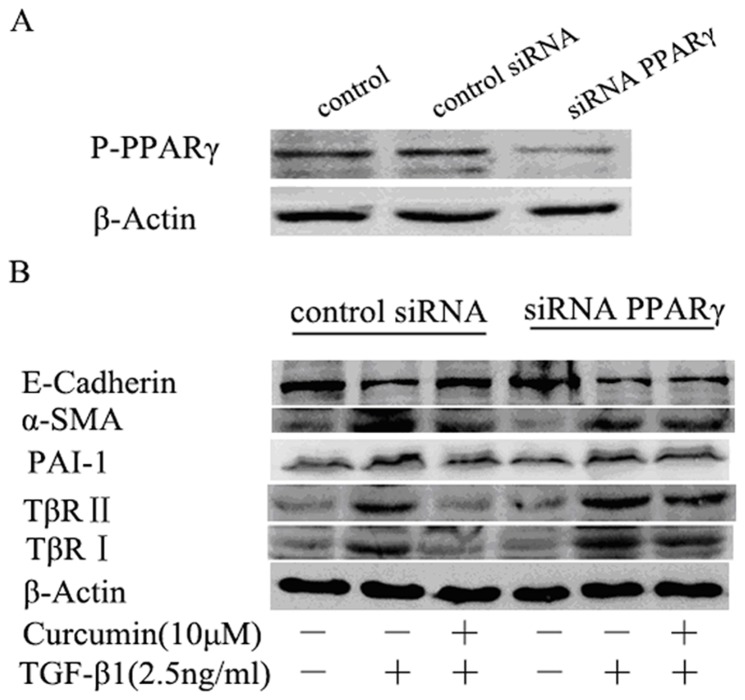
Effect of curcumin on TGF-β1-induced EMT in HK-2 cells is dependent on PPARγ. (A) Knockdown of PPARγ in HK-2 cells. HK-2 cells were infected with PPARγ-specific shRNA or scrambled shRNA for 3 hours and then changed the medium. The expression of PPARγ was examined by western blotting. (B) After infected with PPARγ-specific shRNA or scrambled shRNA for 3 hours, HK-2 cells were treated with curcumin (10 μM) for 12 hours at indicated dose followed by treatment with TGF-β1 (2.5 ng/ml) for 36 hours. Expression of α-SMA, PAI-1, E-cadherin, TβR II and TβR I were detected by western blotting.

Furthermore, we knocked down the expression of PPARγ at cellular level using shRNA interference via lentiviral infection of HK-2 cells. Results from western blotting showed that the PPARγ protein in knocked-down cells was significantly lower compared to that in normal cells and scrambled shRNA infected cells (**Fig.** **8a**). With the significantly decreased PPARγ expression by shRNA the effects of curcumin on α-SMA, PAI-1, E-cadherin, TβR I and TβR II were inhibited (**Fig.** **8b**).

### 8. ERK phoshorylates PPARγ in HK-2 cells

**Figure 9:**
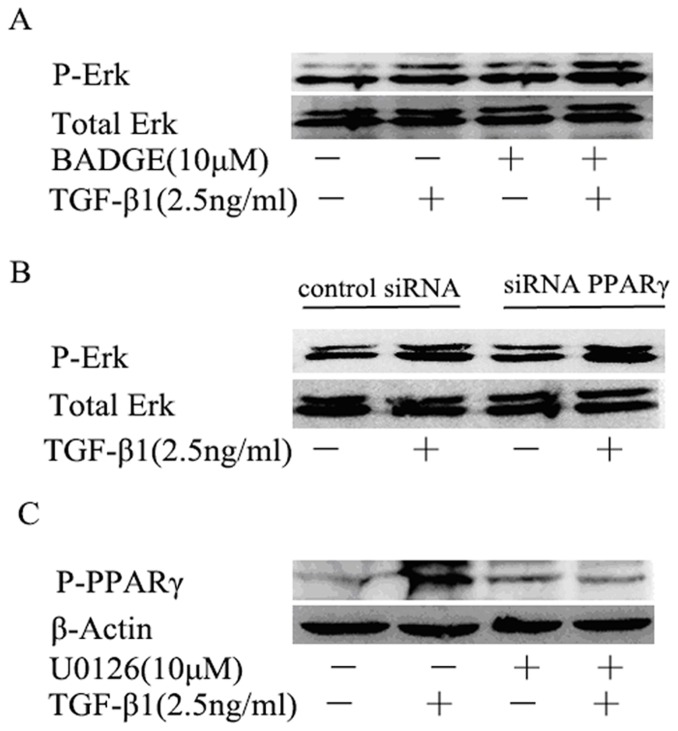
Crosstalk between ERK and PPARγ signaling. (A) Effect of blocking PPARγ signaling by pharmacological inhibitor on phoshorylation of ERK. HK-2 cells were pretreated with BADGE (10 μM) 1 hour before addition of TGF-β1 (2.5 ng/ml) for 30 minutes and the expression of p-ERK were analyzed by western blotting. (B) Effect of knockdown of PPARγ at cellular level on phoshorylation of ERK. HK-2 cells infected with PPARγ-specific shRNA or scrambled shRNA were treated TGF-β1 (2.5 ng/ml) for 30 minutes and the expression of p-ERK were analyzed by western blotting. (C) Effect of blocking ERK signaling on phoshorylation of PPARγ. HK-2 cells were pretreated with U0126 (10 μM) for 1 hour before addition of TGF-β1 (2.5 ng/ml) for 36 hours and the expression of PPARγ were analyzed by western blotting.

Blocking PPARγ signaling pathway by inhibitor BADGE or shRNA (**Fig.** **9a & 9b**) had no effect on the phosphorylation of ERK whereas the suppression of ERK signaling pathway inhibited the phosphorylation of PPARγ (**Fig.** **9c**). These data suggest that ERK signaling pathway is the upstream of PPARγ signaling pathway in the regulation of EMT in HK-2 cells.

### 9. Effect of curcumin on TGF-β1-induced EMT in NRK-52E cells

**Figure 10:**
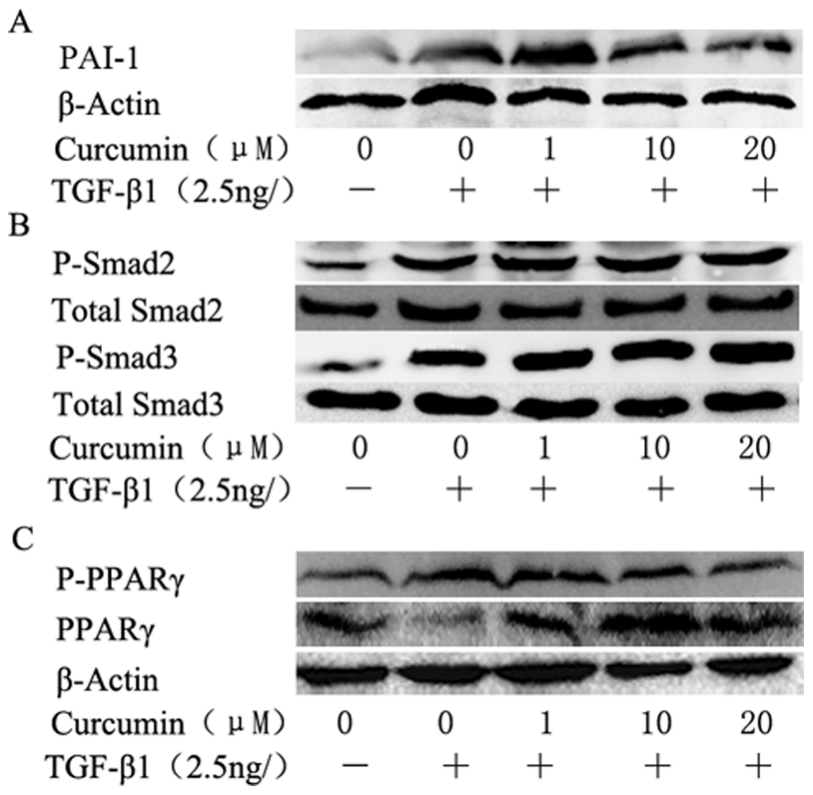
Effects of curcumin on TGF-β1-induced EMT in NRK-52E cells. NRK-52E cells were incubated with curcumin at the indicated dose for 12 hours followed by treatment with TGF-β1 for 36 hours(PAI-1, p-PPARγ and PPARγ) or 30 min(P-Smad2 and P-Smad3). (A) Western blotting analysis for PAI-1 expression. (B) Western blotting analysis for p-Smad2 and p-Smad3 expression. (C) Western blotting analysis for PPARγ and p-PPARγ expression.

Curcumin is also able to block TGF-β1-induced EMT in NRF-52E cells, another renal tubular epithelial cell line (Fig. 10a). As shown in Fig. 10b & 10c, curcumin had no effect on p-Smad2 and p-Smad3, whereas reduced TGF-β1-induced PPARγ phosphorylation and increased the expression of total PPARγ, which is similar to the effects of curcumin on HK-2 cells.

## Discussion

In this study, we investigated the effect of curcumin on TGF-β-induced EMT in HK-2 cells and the underlying mechanism. Increased expression of α-SMA and PAI-1 and decreased expression of E-cadherin are the hallmarks of renal tubular epithelial cells u ndergoing EMT [1], [23], [24]. Suppression of E-cadherin is considered the earliest changes in TGF-β1-induced EMT [25]. Α-SMA is an important marker of fibroblast, contributing to the morphology of the transfered cells and to their ability to migrate and invade. PAI-1, a potent inhibitor of uPA/tPA (urokinase/tissue-type plasminogen activators), plays a major role in extracellular matrix (ECM) accumulation and degradation [26], which is a principal feature in fibrosis. In this study, we observed that with pretreatment of curcumin, the increase of α-SMA and PAI-1 induced by TGF-β1 was attenuated, and the loss of E-cadherin was reversed, confirming the previous reports [17], [27]. These results indicated that curcumin inhibits the process of EMT in renal tubular cell lines.

The mechanisms underlying the effect of curcumin on EMT of HK-2 cells were further investigated. Since binding of TGF-β1 to TβR I and TβR II is the first step of TGF-β signaling [28], we first examined the effect of curcumin on the levels of TβR I and TβR II. Our studies showed that curcumin could inhibit the expression of both TβR I and TβR II, but Gaedeke et al found that curucmin selectively inhibited TβR II in renal fibroblasts cells. It suggested that the signaling might be diffent in tubular and fibroblasts cells.

TGF-β/Smads signaling is considered the most important pathway in EMT and most of the TGF-β-induced EMT appears to be dependent on this signaling. The phosphorylation of Smad2 and Smad3 are essential steps in the signaling cascade. Curcumin was previously demonstrated to suppress the phosphorylation of Smad2 but not that of Smad3 in HK-2 cells [27]. Li et al reported that curcumin inhibited the phosphorylations of both Smad2 and Smad3 in UUO rat model [29]. However, in our study, curcumin failed to affect the phosphorylations of both Smad2 and Smad3 and the expression of total Smad2 and Smad3 in HK-2 cells and NRK-52E cells. The different findings of the phosphorylations of Smad2 and Smad3 by curcumin treatment may be cell type specific, suggesting that the phosphorylation status and effect of Smad2 and Smad3 in EMT are complex and involve many factors.

Besides TGF-β/Smads pathway, TGF-β receptors activate some non-Smad signaling pathways as well, including ERK pathway et al. Studies have demonstrated that ERK is phoshorylated during TGF-β1-induced EMT [30], ERK activity is required for disassembly of adherens junctions and induction of cell motility, and blockade of ERK inhibited key morphological features of EMT in the mammary gland epithelial cells [31], hence the inhibition of phosphorylation of ERK is a potential target for suppressing renal fibrosis. In our study in HK-2 cells, ERK phosphorylation was detected at 5 min after treating with TGF-β1, whereas curcumin attenuated the TGF-β1-induced increase of p-ERK. We pretreated HK-2 cells with U0126, a pharmacological inhibitor of ERK, the inhibitory effect of curcumin on EMT was reduced, indicating that the inhibitory effect of curcumin on EMT is related to its inhibition on ERK signaling.

PPARγ is present and active in multiple renal cell type including the cultured glomerular mesangial cells, podocytes, proximal epithelial cells and epithelial cells of collecting ducts [32], [33]. Studies have shown that PPARγ agonists could ameliorate renal fibrotic lesions in diabetic nephropathy and nondiabetic chronic kidney diseases [34], [35], [36]. In this study, we demonstrated, for the first time, that curcumin induces the expression of PPARγ, inhibits the phosphorylation of PPARγ and promotes the nuclear translocation of PPARγ in renal tubular epithelial cells. Curcumin has been reported to activate PPARγ, but whether it is a ligand is still a debate [19]. The mechanisms by which curcumin induces PPARγ expression remain poorly understood. Two possibilities may be involved: firstly, curcumin binds to its own receptor and the complex stimulates the PPARγ signaling pathway resulting in the up-regulation of PPARγ [19]; secondly, curcumin is a ligand of PPARγ, and binds directly to PPARγ, leading to the activation of PPARγ [18]. Activation of PPARγ by curcumin is important for the inhibition of EMT by curcumin. Down-regulation of PPARγ by its pharmacology inhibitor BADGE or shRNA significantly weakened the inhibition of EMT by curcumin in HK-2 cells, indicating that PPARγ signaling is actively involved in EMT process and curcumin treatment. With both ERK pathway and PPARγ pathway implicated in the mechanism of the inhibition of EMT by curcumin, what is the cross talk between the two pathways in EMT in HK-2 cells? In the inflammatory animal model and cyst formation cell model, studies reported the activating effect of PPARγ by curcumin is attributed to its ability to reduce the intracellular protein p-ERK [37], [38], [39]. But the crosstalk between ERK and PPARγ is still controversial, and it is cell type-dependent. Two possible mechanisms are that either p-ERK phosphorylated and inactivated PPARγ or PPARγ activated ERK [40], [41], [42]. In HK-2 cell, it is very interesting to note in our study that the inhibition of PPARγ by shRNA or BADGE has no effect on the activation of ERK, whereas suppression of ERK using U0126 blocks the phosphorylation of PPARγ. These findings suggest that there is one way communication between ERK and PPARγ, in which ERK affects PPARγ but not in the reverse order in HK-2 cells.

In conclusion, our studies, for the first time, provided evidence for a protective role of curcumin in counteracting TGF-β1-induced EMT in renal tubular epithelial cells via ERK-dependent and then PPARγ-dependent pathway.
